# Novel subgroups of type 2 diabetes and their association with microvascular outcomes in an Asian Indian population: a data-driven cluster analysis: the INSPIRED study

**DOI:** 10.1136/bmjdrc-2020-001506

**Published:** 2020-08-17

**Authors:** Ranjit Mohan Anjana, Viswanathan Baskar, Anand Thakarakkattil Narayanan Nair, Saravanan Jebarani, Moneeza Kalhan Siddiqui, Rajendra Pradeepa, Ranjit Unnikrishnan, Colin Palmer, Ewan Pearson, Viswanathan Mohan

**Affiliations:** 1 Diabetology, Madras Diabetes Research Foundation, Chennai, Tamil Nadu, India; 2 Diabetology, Dr Mohan’s Diabetes Specialities Centre Gopalapuram, Chennai, Tamil Nadu, India; 3 Data Management, Madras Diabetes Research Foundation, Chennai, Tamil Nadu, India; 4 Division of Population Health and Genomics, University of Dundee School of Medicine, Dundee, Dundee, UK; 5 Epidemiology, Madras Diabetes Research Foundation, Chennai, Tamil Nadu, India; 6 Division of Cardiovascular and Diabetes Medicine, University of Dundee, Dundee, Dundee, UK

**Keywords:** diabetes mellitus, type 2, India, diabetes complications

## Abstract

**Introduction:**

Type 2 diabetes is characterized by considerable heterogeneity in its etiopathogenesis and clinical presentation. We aimed to identify clusters of type 2 diabetes in Asian Indians and to look at the clinical implications and outcomes of this clustering.

**Research design and methods:**

From a network of 50 diabetes centers across nine states of India, we selected 19 084 individuals with type 2 diabetes (aged 10–97 years) with diabetes duration of less than 5 years at the time of first clinic visit and performed k-means clustering using the following variables: age at diagnosis, body mass index, waist circumference, glycated hemoglobin, serum triglycerides, serum high-density lipoprotein cholesterol and C peptide (fasting and stimulated). This was then validated in a national epidemiological data set of representative individuals from 15 states across India.

**Results:**

We identified four clusters of patients, differing in phenotypic characteristics as well as disease outcomes: cluster 1 (Severe Insulin Deficient Diabetes, SIDD), cluster 2 (Insulin Resistant Obese Diabetes, IROD), cluster 3 (Combined Insulin Resistant and Deficient Diabetes, CIRDD) and cluster 4 (Mild Age-Related Diabetes, MARD). While SIDD and MARD are similar to clusters reported in other populations, IROD and CIRDD are novel clusters. Cox proportional hazards showed that SIDD had the highest hazards for developing retinopathy, followed by CIRDD, while CIRDD had the highest hazards for kidney disease.

**Conclusions:**

Compared with previously reported clustering, we show two novel subgroups of type 2 diabetes in the Asian Indian population with important implications for prognosis and management. The coexistence of insulin deficiency and insulin resistance seems to be peculiar to the Asian Indian population and is associated with an increased risk of microvascular complications.

**Significance of this study**

**What is already known about this subject?**

- Recently five distinct ‘clusters’ of individuals with diabetes with significantly different characteristics have been identified in a Scandinavian population.
- The unique Asian Indian phenotype predisposes them to young-onset type 2 diabetes (T2D).

**What are the new findings?**

- For the first time in India (and South Asia), clustering was done on 19 084 individuals with T2D using eight clinically relevant variables (age at diagnosis, body mass index, waist circumference, glycated hemoglobin, triglycerides, high-density lipoprotein cholesterol, and C peptide fasting and stimulated).
- Four replicable clusters were identified, two of which were unique to the Asian Indian population.
- The novel cluster ‘Combined Insulin Resistant and Deficient Diabetes’ is of particular importance as it is characterized by difficult-to-control hyperglycemia and increased hazards of kidney disease and retinopathy.

**How might these results change the focus of research or clinical practice?**

- Classifying Asian Indians with T2D into phenotypic clusters provides insights into the pathophysiological processes driving diabetes in this ethnic group, which could help in predicting the risk of complications and in focusing attention to individuals with the highest risk of morbidity and mortality.

## Background

Global estimates suggest that 463 million individuals have diabetes as of 2019 and that this number will increase to 700 million by 2045.^1^ More than 90% of these individuals have type 2 diabetes, a condition that is characterized by considerable heterogeneity in its etiopathogenesis and clinical presentation. This heterogeneity has significant implications on the treatment and prognosis of patients with this condition.

Recently, distinct ‘clusters’ or subgroups of individuals with type 2 diabetes have been identified in a Scandinavian population of 8980 individuals, based on five parameters representing the clinical presentation as well as the presence of insulin resistance and beta-cell dysfunction.^2^ These five subgroups have been termed severe autoimmune diabetes, severe insulin deficient diabetes, severe insulin resistant diabetes, mild obesity-related diabetes and mild age-related diabetes. Further analyses of these subgroups have shown that such clustering might have implications with respect to the risk of diabetes complications as well as selection of the most appropriate treatment. However, as the above study has been performed on a white Caucasian population, there is still no clarity on whether this classification is applicable to individuals with diabetes belonging to other ethnic groups.

Asian Indians (South Asians) represent an ethnic group with high predilection for developing type 2 diabetes; indeed, some of the largest increases in diabetes prevalence have been reported from the South Asian region. Type 2 diabetes in Asian Indians differs from that in white Caucasians in a number of significant ways. They tend to develop diabetes at a younger age and at lower levels of obesity than do white Caucasians. They also tend to progress faster from stages of ‘pre-diabetes’ to frank diabetes than members of other ethnic groups. The ‘Asian Indian phenotype’, characterized by high levels of abdominal fat and increased insulin resistance even at low levels of body mass index (BMI), has been postulated as a reason for this increased propensity to develop type 2 diabetes.^3^ However, recent studies suggest that beta-cell dysfunction occurs quite early and rapidly in Asian Indians.^4^ Type 2 diabetes in Asian Indians therefore appears to have a slightly different pathophysiology, with severe insulin deficiency being the primary defect in contrast to white Caucasians, in whom the main driver of diabetes is obesity and consequent insulin resistance.

It is therefore possible due to the above and the well-known younger age at diagnosis that clusters of type 2 diabetes identified in Asian Indians based on parameters used in the Western population might not behave exactly in the same manner with respect to treatment outcomes and risk of complications. In this paper, we attempt to identify distinct clusters of type 2 diabetes in Asian Indians and to look at the clinical implications and outcomes of this clustering. This study is part of the INdia-Scotland Partnership for pRecision mEdicine in Diabetes (INSPIRED) project.

## Research design and methods

### Study population

Data for this analysis were obtained from the electronic medical records of a tertiary care center for diabetes, which has 50 branches spread across nine states of India. Clinical data of more than 400 000 patients have been captured and stored in the common diabetes electronic medical records (DEMR) system of the center,^5^ which represents one of the largest databases of patients with diabetes in the world. Each patient is provided a unique identification number at the time of their first registration, and clinical, anthropometric and biochemical data are updated in the system at each subsequent visit. Patients undergo a comprehensive evaluation for classification of diabetes, assessment of control and presence of chronic complications at the time of their index visit, and these tests are repeated subsequently at regular intervals based on prespecified protocols.

The following examinations and investigations are performed for every patient during their clinic visits. Height, weight and waist circumference are measured using standardized techniques and the BMI calculated as weight (in kilograms) divided by the square of height (in meters). Blood pressure is recorded to the nearest 2 mm Hg from the right arm in a sitting position with a mercury sphygmomanometer (Diamond Deluxe BP Apparatus, Pune, India).

Blood samples are collected for the measurement of various parameters including fasting and postprandial plasma glucose, lipid profile, kidney and liver function tests, glycated hemoglobin (HbA1c), and C peptide (fasting and stimulated), while Glutamic Acid Decarboxylase (GAD) autoantibodies are measured in a selected subset of patients. Fasting plasma glucose (FPG), serum cholesterol, serum triglycerides and high-density lipoprotein (HDL) cholesterol are measured using Hitachi 912 Autoanalyzer (Hitachi, Mannheim, Germany). Fasting C peptide levels and stimulated (postbreakfast) C peptide levels are estimated by the electrochemiluminescence method on an Elecsys 2010 machine (Hitachi). To obtain the C peptide values, a fasting blood sample is obtained after an overnight fast of at least 8 hours and a postprandial sample after 90 min of a standard South Indian breakfast (above 250 calories).^6^ HbA1c is measured by high-performance liquid chromatography using the Variant machine (Bio-Rad, Hercules, California, USA).

*Diabetes* is diagnosed if the FPG level is ≥126 mg/dL (7.0 mmol/L) and/or 2-hour postload glucose level is ≥200 mg/dL (11.1 mmol/L) and/or if the patient has been prescribed pharmacotherapy for diabetes by a physician.^7^

*Type 2 diabetes* is diagnosed by absence of ketosis, good beta-cell reserve as shown by fasting C peptide assay >0.6 pmol/mL, absence of pancreatic calculi (on abdominal radiograph), and response to oral hypoglycemic agents for at least 2 years.^8^

Assessment of complications is done as follows:

#### Retinopathy

A detailed retinal (fundus) examination is done by both direct and indirect ophthalmoscopy by a retinal specialist. Fundus photography is done using four-field stereo color retinal photography (Model FF 450 plus camera, Carl Zeiss, Jena, Switzerland). An Early Treatment Diabetic Retinopathy Study grading system that has been modified and standardized in other population-based studies is used for the diagnosis of retinopathy.^9 10^

#### Nephropathy

Microalbuminuria is diagnosed if the albumin excretion was between 30 and 299 µg/mg and macroalbuminuria if albumin excretion is ≥300 µg/mg.^11^ Nephropathy is defined as the presence of either microalbuminuria or macroalbuminuria.

Chronic kidney disease (CKD) is defined as an estimated glomerular filtration rate of less than 60 mL/min/1.73m^2^ as calculated by the CKD-Epi formula.

Diabetic kidney disease is defined as the presence of CKD and/or albuminuria.

Homeostasis Model Assessment of beta-cell function (HOMA-B) and insulin resistance (HOMA-IR) are assessed based on C peptide concentrations and plasma glucose using the HOMA calculator (University of Oxford, Oxford, UK).^12^

From the DEMR, we selected 373 000 individuals aged 10–97 years who had a diagnosis of type 2 diabetes. From these, we further selected 55 429 individuals who had baseline data available for the variables of interest, namely age at diagnosis, BMI, waist circumference, HbA1c, serum triglycerides, serum HDL cholesterol, and C peptide (fasting and stimulated). Of these, we selected 20 850 individuals with reported duration of diabetes less than 5 years at first clinic visit (mean 1.74±1.4 years). After merging the HOMA-B and HOMA-IR and removing individuals with HOMA-B >500 and HOMA-IR <20 (n=1512), removing outliers for the remaining variables by the 5SD method (n=188) and excluding individuals who had a positive test for GAD autoantibodies (n=66), we had a final sample size of 19 084.

### Cluster analysis

The k-means clustering method was done with k value of 4 using k-means function (max iteration=10 000) in R V.3.6.0. Cluster forming tendency of the data was validated by the Hopkins statistic value. The optimal number of clusters was determined based on silhouette width. Cluster-wise stability was computed by Jaccard bootstrap method through resampling of the data set 2000 times. A stable cluster generally yields Jaccard similarity index of greater than 0.75.^13^ Cluster analysis was performed on scaled and centered values. Cluster labels were assigned based on the phenotype characteristics of individual cluster mean values of the variables.

Sensitivity analysis was done using three time periods for duration of diabetes <1 year, <3 years, and <5 years. Clustering tendency of the three different baseline diabetes duration data was found to have Hopkins statistic values of 0.19, 0.18, and 0.16, indicating that there were no significant differences in the cluster groups. Reclustering was done on men and women separately to validate the clustering and avoid the sex-dependent stratification effect on the phenotype variables. The minimum silhouette width was similar in both genders (male and female).

### Statistical models

The risk of development of diabetes complications was calculated using Cox regression models with covariate as a cluster label and adjusted for age at diagnosis and sex, after excluding individuals who already had complications at their first visit to the clinic. Cluster-wise time to reaching target goal was analyzed by Cox regression model. Cox proportional hazards assumptions were tested using R V.3.6.0.

#### Validation of clustering in a nationally representative epidemiological data set (ICMR-INDIAB study)

In order to validate the applicability of this clinic-based clustering to the general population, we attempted to replicate the clustering in the data set derived from the nationally representative Indian Council of Medical Research-India Diabetes (ICMR-INDIAB) study. ICMR-INDIAB is a nationwide population-based study on diabetes and related non-communicable diseases being carried out in all 29 states and 2 of the Union Territories of India based on a representative sample of each state. The detailed methodology of ICMR-INDIAB has been published elsewhere.^14^ Data from ICMR-INDIAB on the prevalence of diabetes in 15 states of India have been published.^15^

For purposes of this validation study, we selected 3851 individuals with type 2 diabetes from the INDIAB study population in 15 states of India and clustering was performed using the following six variables: age at diagnosis, BMI, waist circumference, HbA1c, serum triglycerides and serum HDL cholesterol. We excluded C peptide from the analysis as we did not have data on this variable in the population-based sample. After excluding 1521 individuals who did not have data on all our variables of interest as well as 88 outliers (5SD), we had a final sample size of 2204 individuals for the analysis. The k-means clustering was applied with k value as 4 (max iteration=10 000) in R V.3.6.0. The optimal cluster number was determined based on the silhouette width method.

#### Comparison with the Scandinavian clusters

Using the same five variables (age at onset, BMI, HbA1c, HOMA-B and HOMA-IR) reported by Ahlqvist *et al*,^2^ we then attempted to assess whether the clusters identified in the Scandinavian population are replicable in the DEMR-derived Asian Indian clinic population. We applied the k-means clustering (k=4) on the Asian Indian population (N=19 084) based on these five variables and analyzed the optimal cluster number based on the silhouette width method.

We were unable to perform the Scandinavian clustering in the population-based INDIAB sample as we did not have information on variables such as HOMA-B and HOMA-IR in the epidemiological data set.

## Results

Using cluster analysis based on eight clinically relevant variables (age at diagnosis, BMI, waist circumference, HbA1c, serum triglycerides, serum HDL cholesterol, and C peptide fasting and stimulated), we were able to identify four replicable clusters of patients with type 2 diabetes in this Asian Indian population. The optimal number of clusters was based on silhouette width obtained from both the DEMR and the national ICMR-INDIAB data sets (online supplementary figure S1A, B). Table 1 shows the clinical and biochemical characteristics of these four clusters with respect to the eight variables used for clustering and compares other clinically relevant variables across these clusters. The Jaccard similarity index was greater than 0.75, which confirmed that the cluster allocations were stable.

10.1136/bmjdrc-2020-001506.supp1 Supplementary data

**Table 1:** Clinical and biochemical characteristics of the various subgroups of type 2 diabetes

	Cluster 1 (SIDD)	Cluster 2 (IROD)	Cluster 3 (CIRDD)	Cluster 4 (MARD)
n	5009	4934	2313	6828
Frequency, %	26.2	25.9	12.1	35.8
Men, %	65.8	59.8	73.7	58.6
**Age at diagnosis, years**	42.5 (10.8)	46.5 (10.4)	42.1 (9.8)	50.2 (10.3)
**BMI, kg/m^2^**	24.9 (3.5)	32.6 (4.1)	26.5 (3.1)	25.9 (2.9)
**Waist circumference, cm**	90 (8.8)	108 (8.9)	94.9 (8.1)	92.4 (7.4)
**Glycated hemoglobin, %**	10.7 (2.1)	8.3 (1.8)	9.1 (1.9)	7.2 (1.2)
**Glycated hemoglobin, mmol/mol**	93.0	67.0	76.0	55.0
**Serum triglycerides, mg/dL**	149 (59)	155 (59)	351 (102)	136 (50)
**HDL cholesterol, mg/dL**	40 (9)	38 (8)	36 (8)	42 (9)
**C peptide fasting, pmol/mL**	0.8 (0.3)	1.5 (0.4)	1.2 (0.4)	1.1 (0.3)
**C peptide stimulated, pmol/mL**	1.7 (0.6)	3.3 (0.8)	2.6 (0.8)	3 (0.7)
HOMA-B	38.8 (26.9)	100.8 (51.5)	64.5 (37.7)	94.1 (43.1)
HOMA-IR	2.8 (1.6)	4.1 (1.5)	3.8 (1.9)	2.6 (0.8)
WHR	0.93 (0.06)	0.97 (0.08)	0.96 (0.06)	0.94 (0.07)
Systolic blood pressure, mm Hg	123 (16)	128 (16)	127 (17)	127 (17)
Diastolic blood pressure, mm Hg	79 (9)	82 (9)	81 (10)	79 (9)
Serum cholesterol, mg/dL	188 (43)	176 (40)	206 (44)	177 (41)
LDL cholesterol, mg/dL	118 (37)	107 (35)	106 (38)	108 (35)
Insulin at registration, %	24.9	10.6	15.0	5.3
Metformin at registration, %	38.4	71.3	50.9	63.4
Sulfonylureas at registration, %	32.8	44.1	38.0	39.7
Statin at registration, %	30.2	37.8	37.3	37.8
ACE inhibitor at registration, %	2.4	3.5	2.9	3.4

Variables in bold are those used for clustering.

ACE, angiotensin converting enzyme; BMI, body mass index; CIRDD, Combined Insulin Resistant and Deficient Diabetes; HDL, high-density lipoprotein; HOMA-B, homeostasis model assessment of beta-cell function; HOMA-IR, homeostasis model assessment of insulin resistence; IROD, Insulin Resistant Obese Diabetes; LDL, low-density lipoprotein; MARD, Mild Age-Related Diabetes; SIDD, Severe Insulin Deficient Diabetes; WHR, waist hip ratio.

*Cluster 1*, referred to as Severe Insulin Deficient Diabetes (SIDD), included 26.2% of clustered patients and was characterized by the lowest BMI and waist circumference, as well as the lowest C peptide (fasting and stimulated) levels. HOMA-B and HOMA-IR were both low in this cluster. These individuals had the highest HbA1c values and were more likely to be using insulin compared with the other clusters.

*Cluster 2* is a novel cluster which we refer to as Insulin Resistant Obese Diabetes (IROD). This comprised 25.9% of clustered patients. These individuals had the highest BMI and waist circumference and the highest C peptide levels. HOMA-B and HOMA-IR were also the highest for this cluster. Metabolic control was intermediate and individuals were more likely to be on metformin.

*Cluster 3*, another novel group identified in this population, is referred to asCombined Insulin Resistant and Deficient Diabetes (CIRDD) and constitutes 12.1% of the study population. This group was characterized by the lowest age at onset. BMI and waist circumference were intermediate between SIDD and IROD. CIRDD had the highest triglyceride and lowest HDL cholesterol levels of all the four groups. C peptide levels were higher than SIDD, but lower than IROD. HOMA-B and HOMA-IR values were also intermediate between SIDD and IROD, indicating coexistence of insulin deficiency and insulin resistance. Metabolic control tended to be poor; however, only 15% were on insulin.

*Cluster 4*, referred to as Mild Age-Related Diabetes (MARD), represented the most frequent cluster in this population (35.8%) and was characterized by later age at onset than other clusters. They were characterized by the highest HDL cholesterol, fairly preserved C peptide values and the best metabolic control of all the four groups. This group had the least use of insulin.

The characteristics of the clusters did not differ when split by gender (online supplementary table S1, online supplementary figure S2A, B) and duration of diabetes (online supplementary table S2).

10.1136/bmjdrc-2020-001506.supp2 Supplementary data

Online supplementary table S3 shows the clustering without including HbA1c in the model. It was observed that the cluster characteristics are similar even without including HbA1c. In this model, SIDD was the most frequent cluster (32.7%), followed by MARD (31.9%), IROD (21.9%) and CIRDD (13.5%).

Table 2 shows the Cox proportional HR for various microvascular complications of diabetes among the clusters. SIDD had the highest hazards for developing retinopathy, followed by CIRDD (p<0.05), while CIRDD had the highest hazards for kidney disease (both CKD (p<0.05) and proteinuria) after adjusting for age, gender, HbA1c and blood pressure.

**Table 2:** Cox HR for microvascular complications across clusters

	Labels	Events (%)	HR (95% CI)*	P value
Retinopathy	SIDD	4.9	1.56 (1.22 to 1.98)	**<0.001**
	IROD	2.7	0.99 (0.79 to 1.24)	0.95
	CIRDD	4.1	1.31 (1.01 to 1.71)	**<0.05**
	MARD	2.9	1	–
Nephropathy	SIDD	6.4	1.18 (0.96 to 1.45)	0.1094
	IROD	6.3	1.03 (0.87 to 1.23)	0.672
	CIRDD	6.2	1.23 (1.05 to 1.46)	**<0.0001**
	MARD	5.7	1	–
CKD	SIDD	1.5	1.30 (0.94 to 1.78)	0.1031
	IROD	1.8	1.48 (1.12 to 1.97)	**<0.001**
	CIRDD	2.2	2.30 (1.61 to 3.26)	**<0.001**
	MARD	2	1	–
DKD	SIDD	6	1.03 (0.88 to 1.20)	0.7002
	IROD	6.3	1.20 (0.98 to 1.47)	0.0732
	CIRDD	5.7	1.22 (1.03 to 1.45)	**<0.05**
	MARD	5.9	1	–

The bold values denote the differences that have attained statistical significance.

*Adjusted for age, sex, HbA1c and blood pressure, using MARD as the reference group, HR=1.0.

CIRDD, Combined Insulin Resistant and Deficient Diabetes; CKD, chronic kidney disease; DKD, diabetic kidney disease; HbA1c, glycated hemoglobin; IROD, Insulin Resistant Obese Diabetes; MARD, Mild Age-Related Diabetes; SIDD, Severe Insulin Deficient Diabetes.

**Figure 1:**
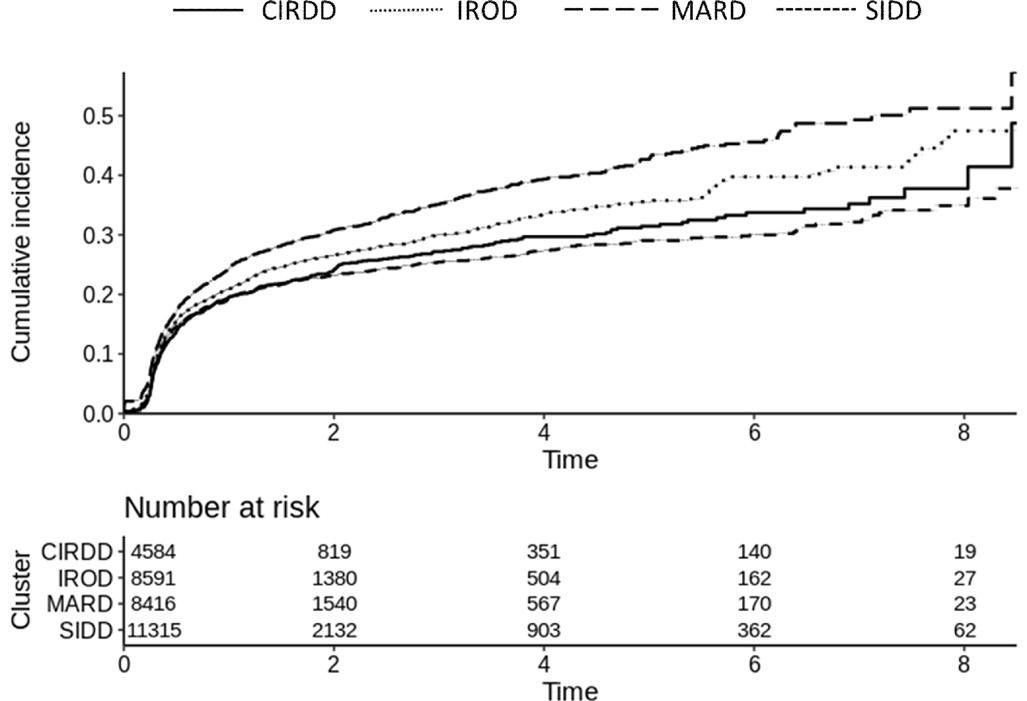
Reaching treatment goal (glycated hemoglobin <7% (53 mmol/mol)). CIRDD, Combined Insulin Resistant and Deficient Diabetes; IROD, Insulin Resistant Obese Diabetes; MARD, Mild Age-Related Diabetes; SIDD, Severe Insulin Deficient Diabetes.

Figure 1 shows the time to reach treatment goal (HbA1c <7% (53 mmol/mol)) for various clusters. MARD showed the shortest time to reach goal, and CIRDD and SIDD the longest (online supplementary table S4).

### Results from the validation with INDIAB data

Results from the validation study with the INDIAB data show that the clusters identified in the clinic population are replicable in this nationally representative data set (table 3).

**Table 3:** Validation of cluster in nationally representative ICMR-INDIAB data (n=2204)

	Cluster 1 (SIDD)	Cluster 2 (IROD)	Cluster 3 (CIRDD)	Cluster 4 (MARD)
n	603	667	167	767
%	27.4	30.3	7.6	34.8
Men, %	54.6	52.0	63.5	57.5
**Age at diagnosis, years**	40.1 (9.8)	48.2 (9.6)	45.4 (10.2)	55.5 (9.8)
**BMI, kg/m^2^**	22.7 (3.1)	29.9 (3.6)	25 (2.9)	23.4 (2.8)
**Waist circumference, cm**	82.8 (9.2)	102.5 (8.0)	90.4 (8.9)	86.1 (8.9)
**Glycated hemoglobin, %**	10.0 (2.1)	7.9 (1.8)	9.0 (2.0)	6.7 (1.2)
**Glycated hemoglobin, mmol/mol**	86.0	63.0	75.0	50.0
**Serum triglycerides, mg/dL**	180.6 (84.0)	187.8 (82.3)	414.0 (48.3)	151.1 (72.8)
**HDL cholesterol, mg/dL**	40.9 (10.5)	37.3 (8.9)	31.6 (8.1)	39.0 (10.3)
Serum cholesterol, mg/dL	183.5 (47.8)	178.3 (41.1)	218.9 (56.6)	171.7 (42.0)
Systolic blood pressure, mm Hg	135.1 (21.3)	141.6 (23.4)	139.6 (21)	142.4 (24.1)
Diastolic blood pressure, mm Hg	82.6 (11.1)	83.7 (12.6)	86 (11.3)	82.2 (12.2)

Variables in bold are those used for clustering.

BMI, body mass index; CIRDD, Combined Insulin Resistant and Deficient Diabetes; HDL, high-density lipoprotein; ICMR-INDIAB, Indian Council of Medical Research-India Diabetes; IROD, Insulin Resistant Obese Diabetes; MARD, Mild Age-Related Diabetes; SIDD, Severe Insulin Deficient Diabetes.

In the INDIAB population, MARD was the most frequent cluster (34.8%), followed by IROD (30.3%), SIDD (24.7%) and CIRDD (7.6%). SIDD had the lowest BMI and waist circumference and highest HbA1c. IROD had the highest BMI and waist circumference and intermediate metabolic control. Individuals in the CIRDD cluster had BMI intermediate between SIDD and IROD, the highest triglycerides and lowest HDL, and high HbA1c and diastolic blood pressure. MARD had the highest age at diagnosis, the highest HDL levels, the lowest diastolic blood pressure and the best metabolic control. In all these respects, the clusters derived from the ICMR-INDIAB population behaved similar to those derived from the DEMR.

### Results of comparison with the Scandinavian clusters

We then undertook the clustering based on the five variables used by Ahlqvist *et al*.^2^ We found that there were considerable differences between the clusters obtained in the Scandinavian population and the Asian Indian population (online supplementary tables S5 and S6). The insulin deficient cluster in the Asian Indian population was similar to SIDD in the Scandinavian population, as was the mild age-related subgroup to MARD (although with a lower age); however, the severely insulin resistant group in our population was also characterized by poor beta-cell function and higher levels of obesity (unlike severe insulin resistant diabetes in the Scandinavian population), while the mild obesity-related diabetes cluster could not be clearly defined in our population.

## Conclusions

In this analysis of around 20 000 individuals with type 2 diabetes from South India, we were able to identify four clusters of patients, differing in phenotypic characteristics as well as disease outcomes with respect to diabetes control and risk of complications. These findings replicated in a population-based study in India across 15 Indian states. Two of these subgroups (SIDD and MARD) correspond to the clusters identified by Ahlqvist *et al*^2^ in the Scandinavian populations, while the other two are novel subgroups (CIRDD and IROD) with certain unique phenotypic and biochemical characteristics.

One of these novel subgroups, which we have termed CIRDD, comprises a minority of patients with type 2 diabetes in our population, but represents a more aggressive phenotype in that the age of onset is lower and their metabolic control is almost as poor as those of the severe insulin deficient (SIDD) group. Also, they took almost as long as those in the SIDD cluster to reach treatment goals. It is likely that the presence of dual pathophysiology renders these individuals at high risk of developing diabetes at younger ages and predisposes them to poorer glycemic control. These individuals also had the highest serum triglyceride levels among all the clusters, possibly on account of accelerated lipolysis, secondary to insulin resistance. The insulin deficiency in these individuals could, in part, be attributed to beta-cell damage due to lipotoxicity. Patients with CIRDD also had the highest hazards of developing kidney disease and the second highest hazards for retinopathy. More aggressive therapy with a combination of agents targeting multiple pathophysiologies of type 2 diabetes may be indicated in these patients (perhaps as early as at the time of diagnosis) so as to help them develop a favorable ‘legacy effect’ and thus help prevent long-term complications. They also need to be screened more aggressively for complications, particularly nephropathy and retinopathy. All these of course have to be tested prospectively through well-planned randomized clinical trials.

Individuals with the second novel subgroup, IROD, had better metabolic control than either SIDD or CIRDD, implying that they have sufficient beta-cell function to at least partially compensate for the obesity-related insulin resistance. However, they also had high risk of developing kidney disease. The higher risk of kidney disease in the two insulin resistant phenotypes (CIRDD and IROD) can be explained by the association of insulin resistance with increased salt sensitivity, glomerular hypertension and hyperfiltration.^16^ The excess risk of kidney disease in CIRDD over and above that in IROD can likely be explained by the poorer glycemic control in the former, on account of concomitant insulin deficiency.

The SIDD phenotype is similar to that described by Ahlqvist *et al*^2^ and had the worst metabolic control and took the longest time to reach treatment goal among the four subgroups. Similar to the Scandinavian population, the risk of retinopathy was highest in this insulin deficient phenotype, underlying the pivotal role played by hyperglycemia secondary to insulin deficiency in the development of this microvascular complication. Enabling timely attainment of glycemic goals in these individuals would require more intensive use of insulin therapy, patient education and adoption of technologies than has been the case thus far.

Individuals in the MARD subgroup formed the most frequent cluster in our population and behaved similar to the corresponding cluster in the Scandinavian population. They had the best metabolic control and the lowest risk of complications of all the four subgroups. However, these individuals had a significantly lower age of onset of diabetes (50.2 years) compared with those in the Scandinavian MARD cluster (67.3 years). This is likely explained by the lower age of onset of diabetes in the Asian Indian population in general. We cannot be certain whether our patients with MARD will continue to exhibit features of a mild phenotype of diabetes for the remainder of their lifespan; regular follow-up and monitoring is therefore essential even in this seemingly benign subgroup of type 2 diabetes.

Recent attempts to replicate the subclassification of type 2 diabetes in the US and Chinese population applying similar variables to those applied in the Scandinavian population have suggested that this European-oriented classification is generalizable to other ethnic groups.^17^ In contrast, when we adopted the same approach in our Asian Indian population, we found that two of the subgroups could not be defined as described by Ahlqvist *et al*.^2^ We postulate that our novel clustering approach (using variables that have been shown to be associated with the Asian Indian phenotype) will be more clinically relevant to our population. While we did use C peptide in our clustering in order to prove the existence of beta-cell deficiency, we were able to replicate the clusters even without the C peptide values. In resource-constrained settings, the use of C peptide may not be feasible. It is therefore gratifying that the model works even without C peptide, which would help to scale up the use of these clusters to smaller clinics in remote areas; however, the predictive accuracy will be slightly lower if this approach is used. Similarly, the cluster characteristics remained stable even if HbA1c was not used, but considering that the clinical accuracy is much improved when HbA1c is used, we prefer that HbA1c stays in the model. Moreover, HbA1c is now part of the standard of care for diabetes in India, as in the rest of the world.

The strengths of our study include the use of a very large database on diabetes and identification of clusters based on phenotypic variables appropriate to the Asian Indian phenotype. However, the study does have a few limitations. Our institution being a private, pay-for-service clinic, data on all the variables of interest were not available for every patient due to financial constraints. Similarly, as our institution is also a tertiary referral center for diabetes, only the more severe or advanced cases of diabetes tend to visit the clinic, and this could have introduced an element of bias into our results. Our results, however, show that these clusters are replicable when applied to a nationally representative population derived from a large epidemiological study, suggesting that they are generalizable to the Asian Indian population with diabetes.

In conclusion, we show that type 2 diabetes in the Asian Indian population can be classified into four phenotypic clusters with important implications for prognosis and management. While two of these clusters correspond to those reported in the white Caucasian population, the other two are novel and unique to the Asian Indian population. Of the four clusters, CIRDD is of particular importance as it represents a more aggressive phenotype of type 2 diabetes characterized by difficult-to-control hyperglycemia and markedly increased hazards of kidney disease and retinopathy. We acknowledge that categorizing patients into subtypes will have less power to predict complications than using the continuous data,^18^ but conceptually we believe it is important to recognize that patients in India differ phenotypically and this phenotypic variation impacts on their risk of complications. Risk prediction either based on allocation to subgroups or using the continuous traits in clinical practice will help physicians tailor their treatment strategies such that individuals receive the most appropriate therapy right from the time of diagnosis.

## References

[1] International Diabetes Federation IDF diabetes atlas. 9 edn Brussels, Belgium, 2019 https://www.diabetesatlas.org

[2] Ahlqvist E, Storm P, Käräjämäki A, et al Novel subgroups of adult-onset diabetes and their association with outcomes: a data-driven cluster analysis of six variables. Lancet Diabetes Endocrinol 2018;6:361–9. 10.1016/S2213-8587(18)30051-2 29503172

[3] Unnikrishnan R, Anjana RM, Mohan V Diabetes in South Asians: is the phenotype different? Diabetes 2014;63:53–5. 10.2337/db13-1592 24357697

[4] Staimez LR, Deepa M, Ali MK, et al Tale of two Indians: heterogeneity in type 2 diabetes pathophysiology. Diabetes Metab Res Rev 2019;35:e3192. 10.1002/dmrr.3192 31145829 PMC6834872

[5] Pradeepa R, Prabu AV, Jebarani S, et al Use of a large diabetes electronic medical record system in India: clinical and research applications. J Diabetes Sci Technol 2011;5:543–52. 10.1177/193229681100500309 21722570 PMC3192621

[6] Snehalatha C, Ramachandran A, Mohan V, et al Pancreatic beta cell response in insulin treated NIDDM patients limitations of a random C-peptide measurement. Diabete Metab 1987;13:27–30. 3552773

[7] Alberti KG, Zimmet PZ, Definition ZPZ Definition, diagnosis and classification of diabetes mellitus and its complications. part 1: diagnosis and classification of diabetes mellitus provisional report of a who consultation. Diabet Med 1998;15:539–53. 10.1002/(SICI)1096-9136(199807)15:7<539::AID-DIA668>3.0.CO;2-S 9686693

[8] Mohan V, Shanthi Rani CS, Amutha A, et al Clinical profile of long-term survivors and nonsurvivors with type 2 diabetes. Diabetes Care 2013;36:2190–7. 10.2337/dc12-1193 23564913 PMC3714469

[9] Early Treatment Diabetic Retinopathy Study Research Group Grading diabetic retinopathy from stereoscopic color fundus photographs: an extension of the modified airlie house classification. Ophthalmology 1991;98:786–806. 10.1016/S0161-6420(13)38012-9 2062513

[10] Rema M, Premkumar S, Anitha B, et al Prevalence of diabetic retinopathy in urban India: the Chennai urban rural epidemiology study (cures) eye study, I. Invest Ophthalmol Vis Sci 2005;46:2328–33. 10.1167/iovs.05-0019 15980218

[11] Unnikrishnan RI, Rema M, Pradeepa R, et al Prevalence and risk factors of diabetic nephropathy in an urban South Indian population: the Chennai urban rural epidemiology study (cures 45). Diabetes Care 2007;30:2019–24. 10.2337/dc06-2554 17488949

[12] Levy JC, Matthews DR, Hermans MP Correct homeostasis model assessment (HOMA) evaluation uses the computer program. Diabetes Care 1998;21:2191–2. 10.2337/diacare.21.12.2191 9839117

[13] Christian H Cluster-wise assessment of cluster stability. Comput Stat Data Anal 2007;52:258–71.

[14] Anjana RM, Pradeepa R, Deepa M, et al The Indian Council of medical Research-India diabetes (ICMR-INDIAB) study: methodological details. J Diabetes Sci Technol 2011;5:906–14. 10.1177/193229681100500413 21880233 PMC3192597

[15] Anjana RM, Deepa M, Pradeepa R, et al Prevalence of diabetes and prediabetes in 15 states of India: results from the ICMR-INDIAB population-based cross-sectional study. Lancet Diabetes Endocrinol 2017;5:585–96. 10.1016/S2213-8587(17)30174-2 28601585

[16] Spoto B, Pisano A, Zoccali C Insulin resistance in chronic kidney disease: a systematic review. Am J Physiol Renal Physiol 2016;311:F1087–108. 10.1152/ajprenal.00340.2016 27707707

[17] Zou X, Zhou X, Zhu Z, et al Novel subgroups of patients with adult-onset diabetes in Chinese and US populations. Lancet Diabetes Endocrinol 2019;7:9–11. 10.1016/S2213-8587(18)30316-4 30577891

[18] Dennis JM, Shields BM, Henley WE, et al Disease progression and treatment response in data-driven subgroups of type 2 diabetes compared with models based on simple clinical features: an analysis using clinical trial data. Lancet Diabetes Endocrinol 2019;7:442–51. 10.1016/S2213-8587(19)30087-7 31047901 PMC6520497

