# Supplementary material for: Novel subgroups of type 2 diabetes and their association with microvascular outcomes in an Asian Indian population: a data-driven cluster analysis: the INSPIRED study
Source: BMJ Open Diabetes Res Care. 2020 Aug 17;8(1):e001506. doi: 10.1136/bmjdrc-2020-001506 (PMC7437708; doi:10.1136/bmjdrc-2020-001506)
Supplement: Supplementary data [file bmjdrc-2020-001506supp001.pdf]

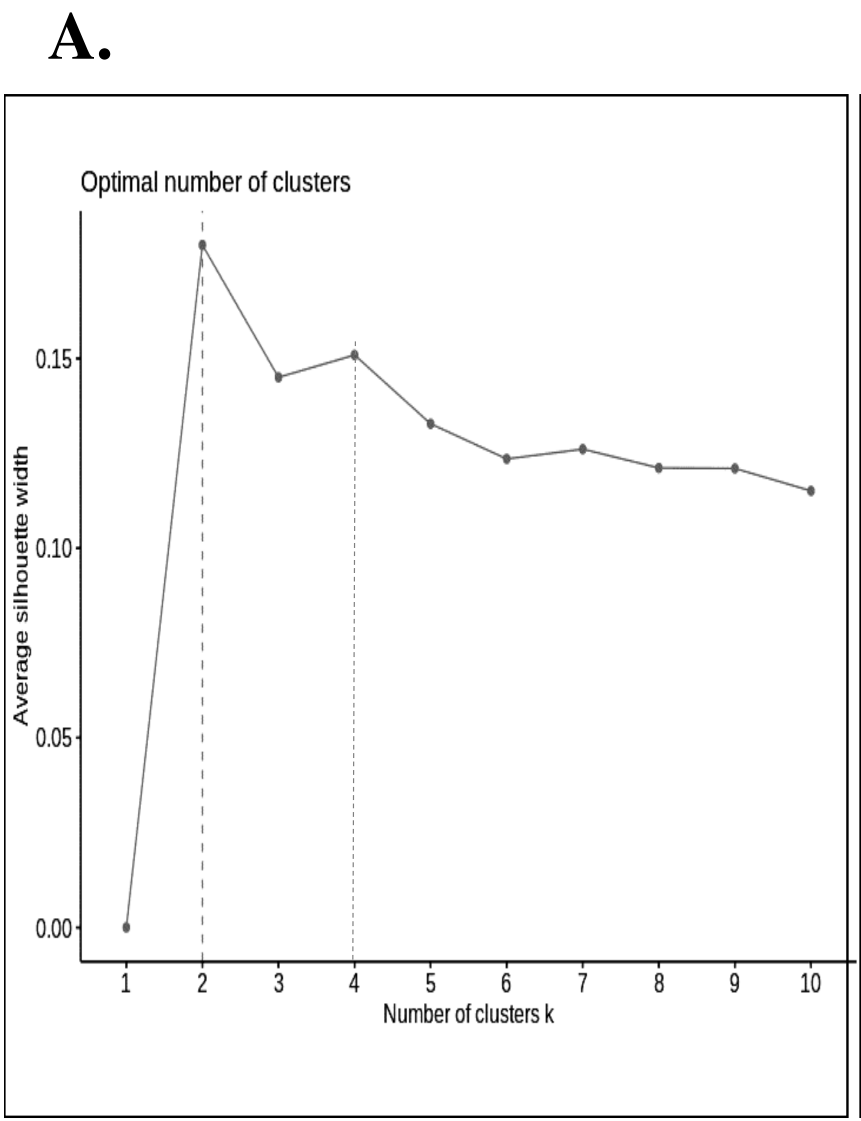

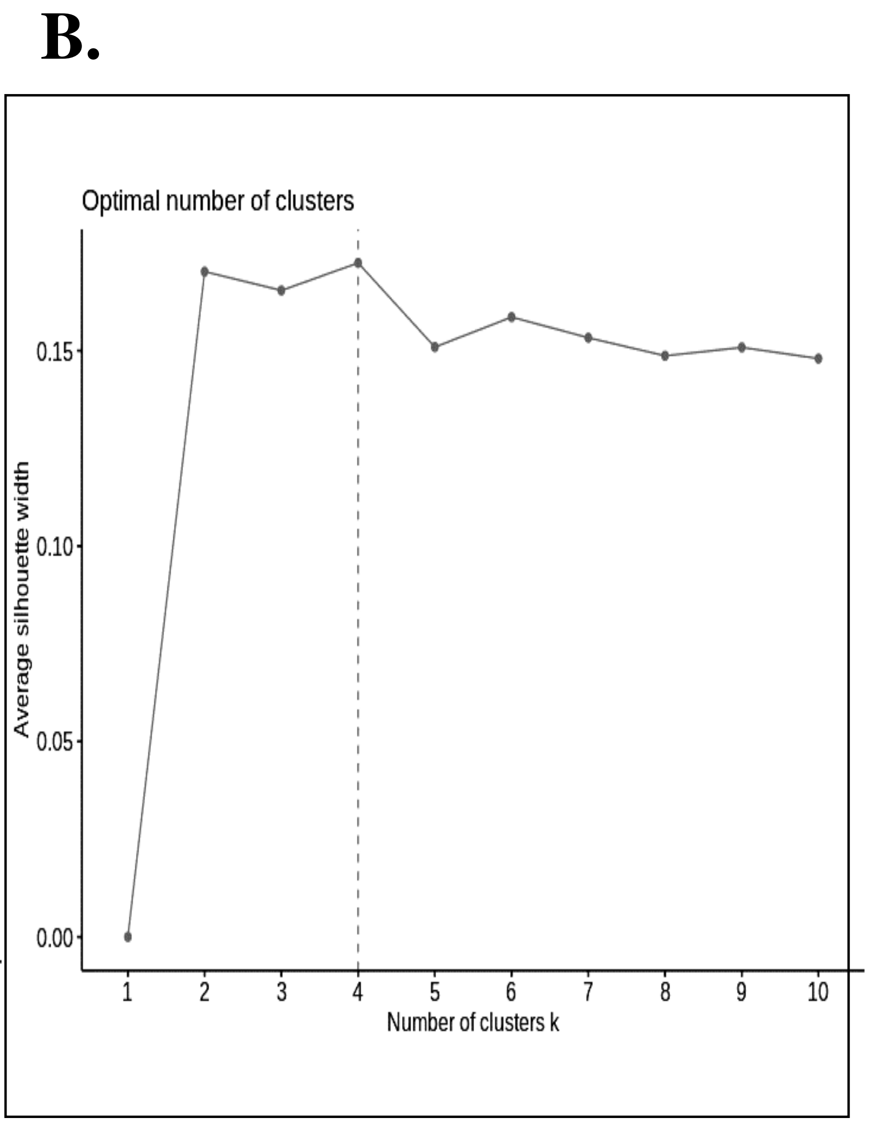

Supplemental Figure S1. Optimal number of clusters based on silhouette width (A) DEMR (B) INDIAB Study

BMJ Publishing Group Limited (BMJ) disclaims all liability and responsibility arising from any reliance placed on this supplemental material which has been supplied by the author(s)

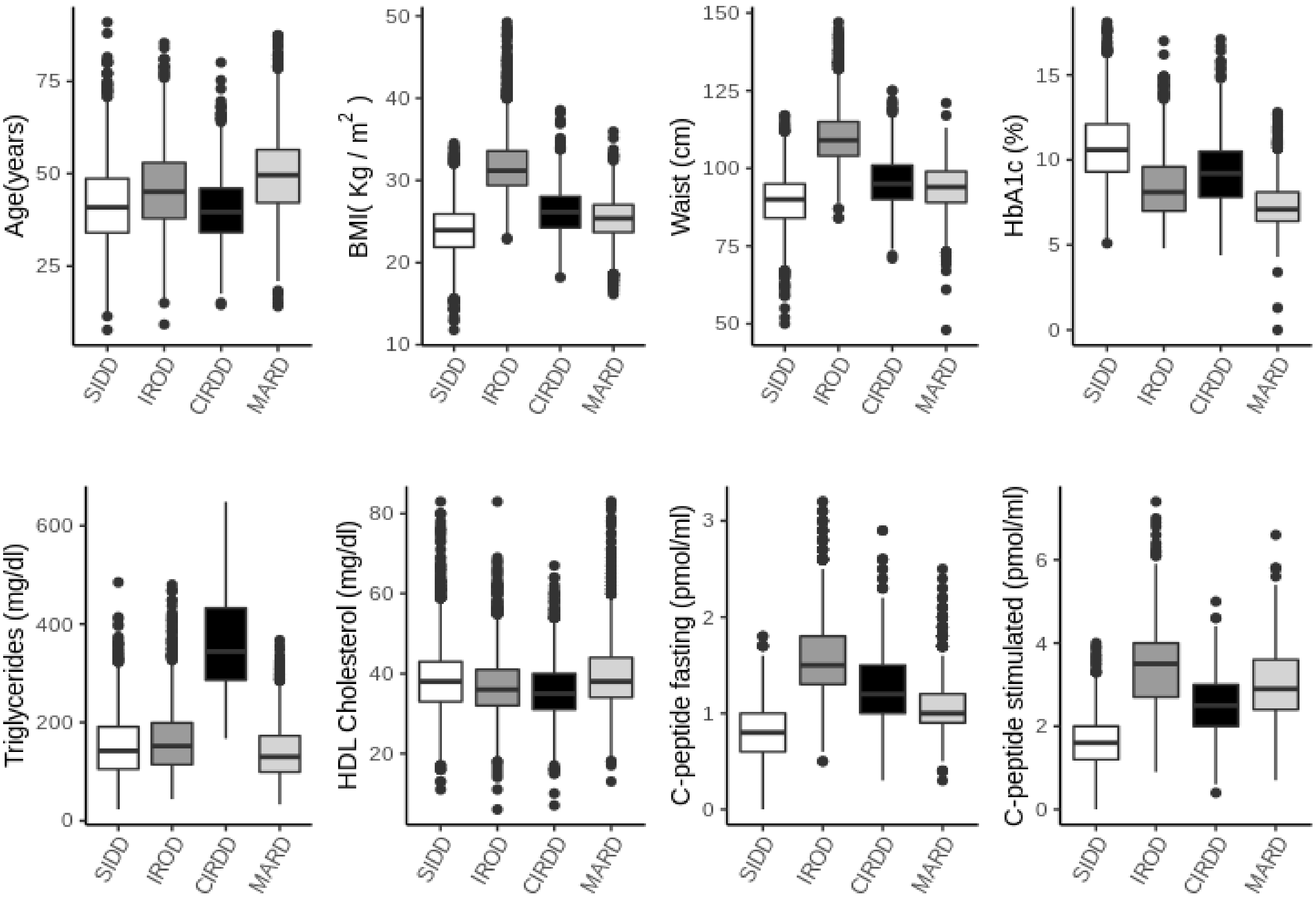

Supplemental Figure S2a. Male patient cluster characteristics

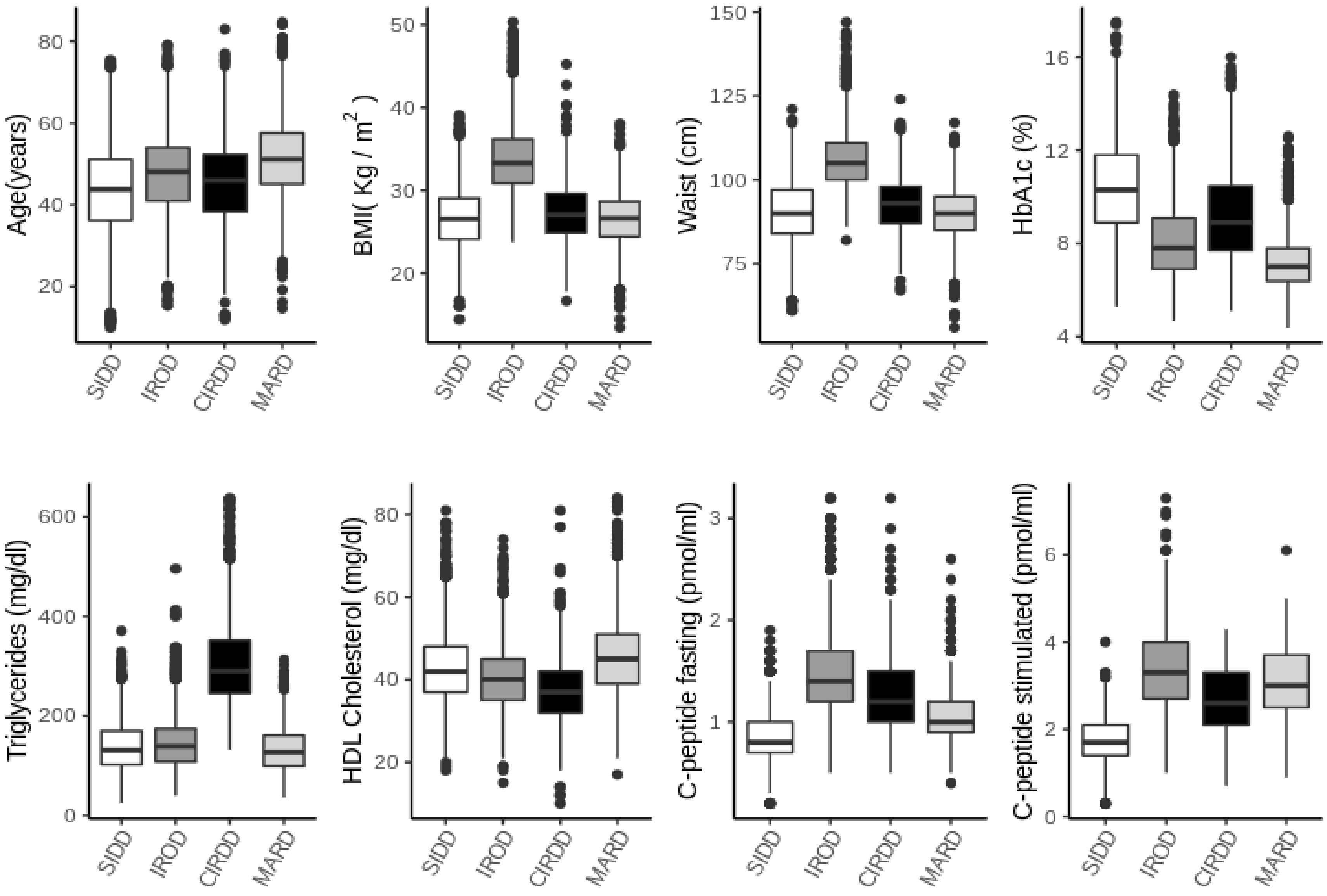

Supplemental FigureS2b. Female patient cluster characteristics

Legend to figures:

Supplemental Figure S1. Optimal number of clusters based on silhouette width. (A)

DEMR. (B) INDIAB Study

Supplemental Figure S2a. Male patient cluster characteristics

Supplemental Figure S2b. Female patient cluster characteristics
