# Supplementary material for: Novel subgroups of type 2 diabetes and their association with microvascular outcomes in an Asian Indian population: a data-driven cluster analysis: the INSPIRED study
Source: BMJ Open Diabetes Res Care. 2020 Aug 17;8(1):e001506. doi: 10.1136/bmjdrc-2020-001506 (PMC7437708; doi:10.1136/bmjdrc-2020-001506)
Supplement: Supplementary data [file bmjdrc-2020-001506supp002.pdf]

Supplemental Table S1 : Cluster-wise phenotype characteristics of male and female patient group

	Cluster 1 (SIDD)		Cluster 2 (IROD)		Cluster 3 (CIRDD)		Cluster 4 (MARD)	
Gender	**Male**	**Female**	**Male**	**Female**	**Male**	**Female**	**Male**	**Female**
N	3116	1910	2921	1971	1472	902	4439	2353
Frequency, %	26.1	26.8	24.4	27.6	12.3	12.6	37.2	33
**Age at diagnosis, years**	41.8 (10.5)	43.5 (11.1)	45.6 (10.6)	47.6 (10)	40.6 (9)	45.4 (10.6)	49.7 (10.5)	51.3 (9.9)
**BMI, kg/m^2**	24 (3.1)	26.6 (3.7)	31.8 (3.7)	33.9 (4.3)	26.2 (2.9)	27.3 (3.6)	25.3 (2.5)	26.6 (3.2)
**Waist Circumference, cm**	89.5 (8.4)	90.4 (9.1)	109.8 (8.6)	105.9 (8.8)	95.6 (7.7)	92.8 (8.2)	94.0 (6.8)	89.9 (7.6)
**Glycated hemoglobin, %**	10.7 (2.1)	10.4 (2.1)	8.4 (1.8)	8.1 (1.7)	9.2 (1.9)	9.2 (2.0)	7.3 (1.3)	7.2 (1.2)
**Glycated hemoglobin, mmol/mol**	93.0	90.0	68.0	65.0	77.0	77.0	56.0	55.0
**Serum triglycerides, mg/dl**	153.1 (62.7)	138.2 (50.3)	162.4 (65.8)	145.7 (51.3)	367.1 (105.3)	308.3 (92.2)	139.7 (53.8)	132.8 (45.8)
**HDL Cholesterol, mg/dl**	38.7 (8.5)	42.9 (9.1)	36.8 (7.3)	40.3 (8)	35.7 (7.4)	37.7 (7.8)	39.3 (8.2)	45.6 (9.1)
**C-peptide fasting, pmol/ml**	0.8 (0.3)	0.8 (0.3)	1.5 (0.4)	1.5 (0.4)	1.2 (0.4)	1.3 (0.4)	1.1 (0.3)	1.1 (0.3)
**C-peptide stimulated, pmol/ml**	1.6 (0.6)	1.7 (0.5)	3.3 (0.8)	3.3 (0.8)	2.5 (0.8)	2.7 (0.8)	3 (0.7)	3.1 (0.7)
HOMA-B	38.2 (26.5)	40.9 (26.8)	101 (53)	99.7 (48.7)	62.6 (37.4)	68.2 (40.4)	93.8 (43.8)	95.4 (42.8)
HOMA-IR	2.7 (1.5)	2.8 (1.7)	4.2 (1.6)	3.9 (1.4)	3.8 (1.6)	3.9 (2.5)	2.7 (0.9)	2.6 (0.8)
Serum cholesterol, mg/dl	186.5 (43.3)	191 (42.3)	175.7 (39.6)	177.3 (39)	206.9 (42.8)	206.5 (46.3)	172.6 (40.3)	183.6 (41)
Insulin at registration, %	28.0	20.4	12.7	8.1	16.0	12.6	5.4	4.9
Metformin at registration, %	41.2	34.6	77.4	65.8	58.3	35.4	56.4	73.9
Sulfonylureas at registration,%	35.7	28.7	48.5	39.8	42.9	27.6	36.0	45.1

Note: * Variables in bold are those used for clustering

Supplemental Table S2 : Characteristics of type 2 diabetes sub-groups with < 1 year and < 3 years of diabetes duration

	Cluster 1 (SIDD)		Cluster 2 (IROD)		Cluster 3 (CIRDD)		Cluster 4 (MARD)	
Duration of diabetes	**< 1 year**	**< 3 years**	**< 1 year**	**< 3 years**	**< 1 year**	**< 3 years**	**< 1 year**	**< 3 years**
N	1883	3379	1637	3305	702	1531	2283	4644
Frequency, %	28.9	26.3	25.2	25.7	10.8	11.9	35.1	36.1
Men, %	66	66.4	58.7	59.3	74.4	74.7	58.5	57.4
Age at diagnosis, years	42.2 (11.9)	42.5(11.4)	46 (11.9)	46.3(10.7)	41.5 (10.5)	42(10.2)	51 (11.1)	50.4(10.5)
BMI, kg/m^2	24.8 (3.4)	24.9(3.5)	32.7 (3.4)	32.7(4.2)	26.6 (3.2)	26.5(3.2)	25.7 (2.9)	25.9(3.0)
Waist Circumference, cm	89.5 (8.6)	89.8(8.8)	107.7 (8.6)	108.1(8.9)	94.5 (8.2)	94.8(8.2)	92 (7.4)	92.3(7.5)
Glycated hemoglobin, %	11.3 (2.1)	10.9(2.1)	8.6 (2.1)	8.3(1.8)	10.0 (2.2)	9.3(2)	7.5 (1.5)	7.3(1.3)
Glycated hemoglobin, mmol/mol	100.0	96.0	70.0	67.0	86.0	78.0	58.0	56.0
Serum triglycerides, mg/dl	147.6 (55.6)	148.3(58.8)	157.1 (55.6)	155.1(59.6)	364.7 (104.5)	350.2(102.3)	135.1 (51.5)	135.5(50.1)
HDL Cholesterol, mg/dl	39.3 (8.7)	39.7(8.8)	37.9 (8.7)	37.9(7.7)	36.3 (7.8)	36(7.4)	41.9 (9.1)	42.1(9.0)
C-peptide fasting, pmol/ml	0.8 (0.3)	0.8(0.3)	1.5 (0.3)	1.5(0.4)	1.2 (0.4)	1.2(0.4)	1.1 (0.3)	1.1(0.3)
C-peptide stimulated, pmol/ml	1.6 (0.6)	1.7(0.6)	3.3 (0.6)	3.3(0.8)	2.5 (0.8)	2.6(0.8)	3.0 (0.7)	3.0(0.7)
HOMA-B	38.5 (27.7)	39.0 (27.4)	101.2 (27.7)	102.8(52.5)	57.7 (36.2)	64.2(38)	94.8 (44.2)	94.9(42.9)
HOMA-IR	2.8 (1.5)	2.8(1.6)	4.2 (1.5)	4.1(1.6)	3.9 (2.1)	3.8(1.8)	2.7 (0.9)	2.7(0.8)
Serum cholesterol, mg/dl	190 (43.4)	188.8(43)	181.3 (43.4)	177.7(39.9)	214.4 (46.4)	207.6(44.1)	179.1 (42.5)	178.4(41.3)

Supplemental Table S3: Validation of cluster without including HbA1c in the model

	Cluster 1 (SIDD)	Cluster 2 (IROD)	Cluster 3 (CIRDD)	Cluster (MARD)
N	6250	4171	2574	6089
Frequency, %	32.7	21.9	13.5	31.9
Men, %	64.5	60.0	74.2	64.5
**Age at diagnosis, years**	42.5 (10.1)	44.8 (10.0)	41.5 (9.4)	53.1 (9.6)
**BMI, kg/m^2**	24.6 (3.3)	33.4 (3.9)	26.5 (3.0)	26.7 (2.9)
**Waist Circumference, cm**	89 (8.2)	109.6 (8.6)	94.8 (7.8)	94.7 (7.1)
**Serum triglycerides, mg/dl**	138.9 (53.7)	156.8 (61.8)	339.1 (103.1)	141 (50.9)
**HDL Cholesterol, mg/dl**	40.8 (9.1)	37.8 (7.6)	35.9 (7.4)	41.6 (8.9)
**C-peptide fasting, pmol/ml**	0.8 (0.2)	1.5 (0.4)	1.2 (0.3)	1.2 (0.3)
**C-peptide stimulated, pmol/ml**	1.8 (0.6)	3.2 (0.8)	2.5 (0.8)	3.2 (0.7)
HOMA-B	51.6 (35.3)	98.1 (51.6)	64.5 (39.5)	96.1 (47.4)
HOMA-IR	2.4 (1.3)	4.1 (1.6)	3.7 (1.8)	3.1 (1.2)
Glycated hemoglobin, %	9.4 (2.4)	8.4 (1.8)	9.2 (2.1)	7.9 (1.8)
Serum cholesterol, mg/dl	182.7 (42.5)	176.2 (39.6)	204 (43.7)	179.9 (42)

Note: *Variables in bold are those used for clustering

Supplementary Table S4: Cox hazards ratio for reaching target goal

Label	%	**HR(CI-95%)***	**P**
SIDD	26	0.69 (0.64 – 0.75)	**<0.001**
IROD	22.6	0.83 (0.76 – 0.90)	**<0.001**
CIRDD	23.7	0.73 (0.66 – 0.80)	**<0.001**
MARD	19.5	1	-

* adjusted for age, following MARD as the reference group, HR = 1.0

Supplemental Table S5 : Patient characteristics of Scandinavian population, an outcome of Ahlqvist et al clustering

	SIDD	SIRD	MOD	MARD
N	1575	1373	1942	3513
Frequency, %	17.5	15.3	21.6	39.1
Men, %	64.8	58.8	52.1	62.0
BMI, kg/m^2	28.86(4.77)	33.85(5.24)	35.71(5.43)	27.94(3.44)
Age at diagnosis, years	56.74(11.14)	65.25(9.34)	48.96(9.54)	67.37(8.55)
Glycated hemoglobin, %	11.5 (3.9)	7.1(3.6)	7.4 (3.6)	6.7 (3.1)
Glycated hemoglobin, mmol/mol	102.0	54.0	57.0	50.0
HOMA-B	47.64(28.93)	150.47(47.20)	95.03(32.45)	86.59(26.37)
HOMA-IR	3.18 (1.73)	5.54 (2.74)	3.35 (1.21)	2.55 (0.84)

Supplemental Table S6: Patient characteristics of Asian Indian population applying Ahlqvist et al clustering phenotypes

	**SIDD**	**SIRD**	**MOD**	**MARD**
**N**	4836	3219	7280	3749
**Frequency, %**	25.3	16.9	38.1	19.6
**Men, %**	69.1	54.9	64.5	57.2
**BMI, kg/m^2**	24.9 (3.1)	32.4 (4.5)	25.5 (3)	30.1 (4.3)
**Age at diagnosis, years**	41.9 (10.7)	44.1 (10.7)	48 (10.4)	50.2 (10.1)
**Glycated hemoglobin, %**	11.1 (1.7)	9.8 (1.7)	7.4 (1.1)	6.9 (1.1)
**Glycated hemoglobin, mmol/mol**	98.0	84.0	57.0	52.0
**HOMA-B**	35.5 (19.9)	60.6 (27.4)	76.9 (26)	148.4 (44.7)
**HOMA-IR**	2.9 (1.0)	4.9 (2.4)	2.5 (0.8)	3.3 (1.0)
Waist Circumference, cm	90.8 (8.6)	105.9 (10.5)	92.4 (8.4)	101.7 (10.3)
Serum triglycerides, mg/dl	184.5 (102.2)	193.3 (101.8)	160.7 (85.7)	151.8 (74.1)
HDL Cholesterol, mg/dl	39.6 (8.9)	38.3 (8.2)	40.6 (8.9)	39.5 (8.5)
C-peptide fasting, pmol/ml	0.9 (0.3)	1.5 (0.4)	1.0 (0.3)	1.4 (0.4)
C-peptide stimulated, pmol/ml	1.9 (0.7)	2.9 (0.8)	2.7 (0.8)	3.5 (0.7)
Serum cholesterol, mg/dl	194.1 (44.1)	188.2 (42.6)	179.9 (41.2)	171.4 (39.9)

Note: * Variables in bold are those used for clustering
